# Advances in PLGA-based polymeric nanocarriers for colorectal cancer therapy: overcoming chemoresistance through controlled delivery strategies

**DOI:** 10.1186/s12943-025-02435-2

**Published:** 2025-11-13

**Authors:** Mohammad Sakib Khan, Urushi Rehman, Taha Alqahtani, Humood Al Shmrany, Garima Gupta, Khang Wen Goh, Amirhossein Sahebkar, Prashant Kesharwani

**Affiliations:** 1 Department of Pharmaceutics, School of Pharmaceutical Education and Research, Jamia Hamdard, New Delhi, 110062 India; 2 Department of Pharmacology, College of Pharmacy, King Khalid University, Abha, 62529 Saudi Arabia; 3 Department of Medical Laboratory, College of Applied Medical Sciences, Prince Sattam Bin Abdulaziz University, Alkharj, 11942 Saudi Arabia; 4 Graphic Era Hill University, Dehradun, 248002 India; 5 School of Allied Medical Sciences, Lovely Professional University, Phagwara, Punjab India; 6 Faculty of Data Science and Information Technology, INTI International University, Nilai, Malaysia; 7 Biotechnology Research Center, Pharmaceutical Technology Institute, Mashhad University of Medical Sciences, Mashhad, Iran; 8 Applied Biomedical Research Center, Basic Sciences Research Institute, Mashhad University of Medical Sciences, Mashhad, Iran; 9 Department of Pharmaceutical Sciences, Dr. Harisingh Gour Vishwavidyalaya (A Central University), Sagar, Madhya Pradesh 470003 India

**Keywords:** Poly(lactic-co-glycolic acid), Polymeric nanocarriers, Colorectal cancer, Chemoresistance, Controlled drug release, Targeted therapy

## Abstract

Colorectal cancer (CRC) is among the most prevalent malignancies globally, with a notably higher incidence in men. Despite the availability of multiple chemotherapeutic options, clinical efficacy remains limited due to poor drug solubility, restricted intestinal absorption, rapid systemic clearance, and multidrug resistance. Poly(lactic-co-glycolic acid) (PLGA), a well-established biodegradable and biocompatible copolymer, has gained significant attention for its potential in developing advanced drug delivery systems. Its tuneable degradation kinetics governed by the lactic-to-glycolic acid ratio enable precise modulation of drug release profiles. PLGA-based nanocarriers offer several therapeutic advantages, including improved solubility, extended colonic retention, and targeted delivery of anticancer agents. Furthermore, surface functionalization with copolymers, ligands, or stimuli-responsive moieties has demonstrated enhanced cellular uptake, tumor specificity, and cytotoxicity against CRC cells. This review highlights the recent developments in the synthesis, functional design, and biomedical application of PLGA nanocarriers in CRC treatment, emphasizing their role in overcoming key challenges such as chemoresistance and off-target toxicity. These advancements underscore the potential of PLGA-based polymeric systems in enhancing the therapeutic index and translational viability of anticancer therapies.

**Graphical abstract:**

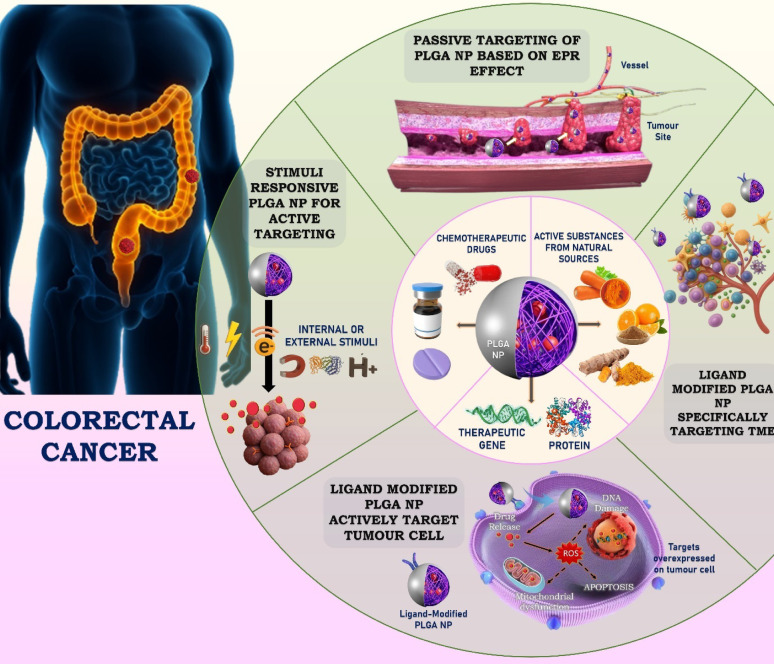

## Introduction

**Fig. 1:**
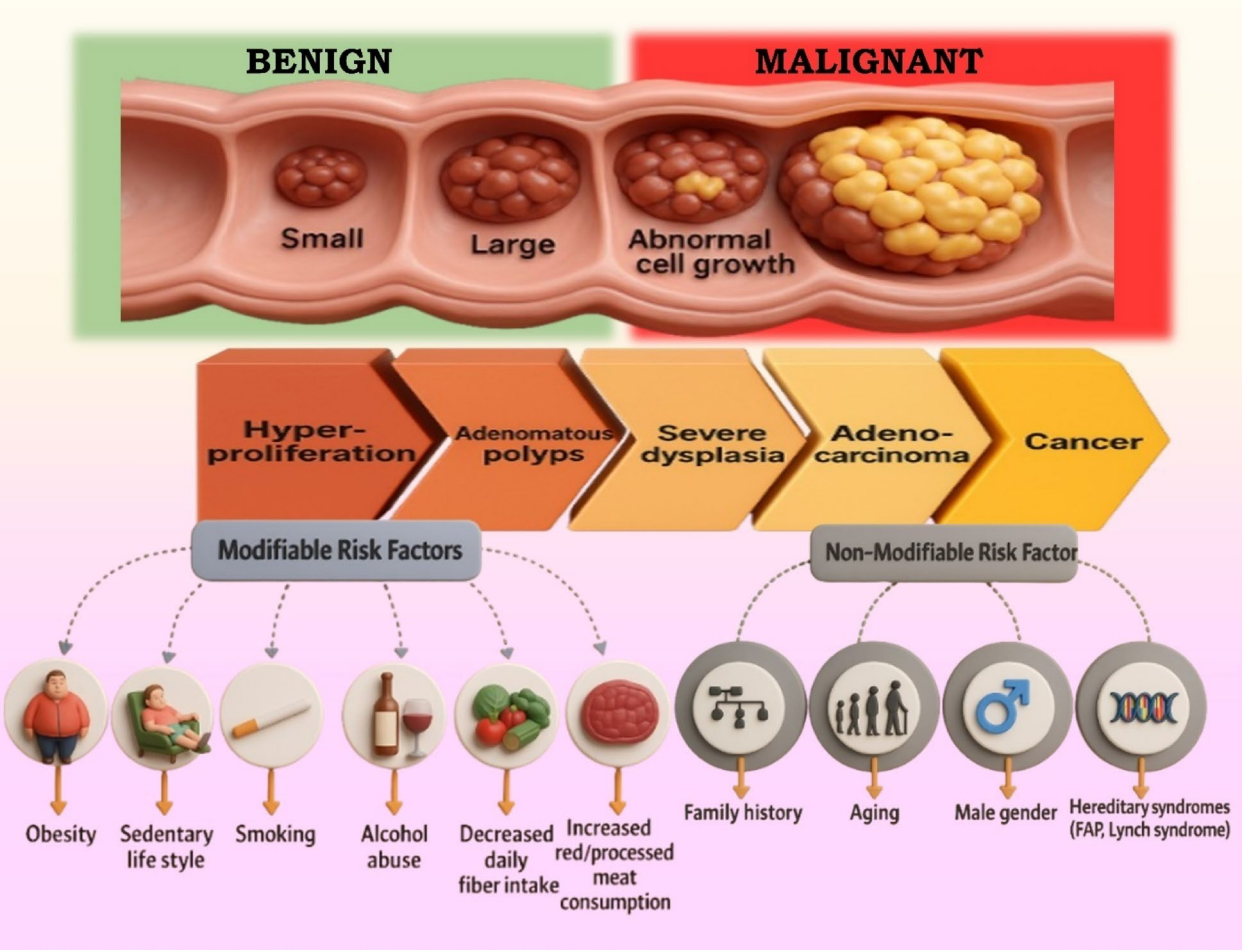
Progression of colorectal cancer from benign to malignant stages, highlighting key modifiable and non-modifiable risk factors influencing its development

Colorectal cancer (CRC) ranks as the third most prevalent cancer globally, following prostate and lung cancers in men and breast and lung cancers in women. In 2022, the global incidence surpassed 1.9 million cases, comprising 1,045,413 in males and 826,706 in females, with an age-standardized rate of 17.8 per 100,000. CRC contributes to approximately 9% of all cancer-related deaths, with notably higher incidence in industrialized nations and rising trends in developing regions due to urbanization, lifestyle changes, and aging populations. This global burden underscores the urgency for effective and targeted treatment modalities (Fig. 1) [1]. Surgical excision is the main treatment for CRC that can be resected; while immunotherapy, chemotherapy, and radiation therapy are common treatments for CRC that cannot be resected. Nevertheless, these treatments have certain disadvantages, including the fact that they are cytotoxic to healthy cells and non-specific, which might result in secondary problems [2]. These treatments can be used in combination, depending on the confinement and advancement of the CRC. Despite this, over 50% patients develop acquired multidrug-resistant CRC [3]. Developing novel CRC therapies to overcome the aforementioned challenges is essential.

With its ability to deliver targeted chemotherapy directly and selectively to cancer cells, nanotechnology is a key component of cancer treatment. Additionally, when compared to conventional chemotherapies, nanotechnology improves the likelihood of patient survival [4]. Colloidal particles of nanometre size (less than 100 nm) that include a therapeutic substance in their matrix are called nanoparticles (NPs). Their sub-micron size allows them to penetrate deep tissue, pass through epithelial fenestrations, and be efficiently absorbed by target cells, all of which increase the bioavailability of therapeutic agents [5]. It is also possible to optimize the active moiety’s release rate and extent by adjusting the properties of the particle polymer. Bio-specific ligand-drug conjugates improve cell targeting and tissue-specific delivery further [6, 7].

Poly (lactic-co-glycolic acid) (PLGA) stands as the sole biodegradable polymer recognized and approved by both the US Food and Drug Administration (FDA) and the European Medicines Quality Agency (EMA) [8]. A variety of active therapeutic elements including proteins, peptides, and small compounds, can be released over a period of weeks to months using PLGA NPs. It overcomes the drawbacks of chemotherapeutic drug therapy to offer a range of surface alterations, attain high load capacity, and shield them from degradation [9].

This review examines the function of PLGA NPs in the treatment of CRC. We begin by describing the chemical makeup, biodegradation, and advantageous characteristics of PLGA, including its regulatory approval and biocompatibility. The next section deals with the active and passive tumour-targeting mechanisms. It then delves into the recent development in CRC treatment strategies involving PLGA. In our view, the unique versatility of PLGA positions it as an ideal platform to tackle both chemoresistance and bioavailability issues in colorectal cancer.

## PLGA nanoparticles: properties and advantages

PLGA is a synthetic biodegradable polymer used for targeted medication delivery. It breaks down into endogenous monomers with minimal toxicity [10, 11]. During biodegradation, lactic and glycolic acids are released, which are removed through the Krebs cycle. Its relatively rapid biodegradation prevents long-term polymer accumulation, making it preferable for chronic CRC therapies [12, 13]. PLGA’s flexibility in polymer ratio (lactic: glycolic acid), molecular weight, and particle size allows for precise control of drug release kinetics critical for overcoming drug resistance in CRC [14]. For instance, PLGA nanoparticles can be tailored to maintain effective concentrations of 5-fluorouracil (5-FU) or camptothecin, protecting them from rapid enzymatic degradation and reducing the risk of recurrence from subtherapeutic dosing [15]. Multiple fabrication methods such as solvent precipitation, emulsion-solvent evaporation, and spray drying allow for encapsulation of fragile payloads including nucleic acids, chemotherapeutics, or immune modulators, often used in CRC treatment strategies (Table 1). These methods also support co-delivery platforms essential for combination regimens targeting CRC chemoresistance [13].

**Table 1:** Comparative insights into nanoparticle preparation techniques, balancing innovation with practical challenges

Preparation method	Key characteristics	Advantages	Disadvantages
Emulsion-solvent evaporation	Produces particles with controllable degradation rates	Controlled degradation profile allows sustained drug release	Degradation rate can vary with environmental pH and temperature
Spray drying	Yields crystalline particles; suitable for dry powders	Enhances particle stability and shelf life	High crystallinity may hinder rapid drug release
Solvent precipitation	Yields uniform particle size	Offers nanoparticles of uniform size for predictable behavior in vivo	May require high energy inputs and solvent removal processes
Single and double emulsion methods	Enables surface modification with ligands/polymers	Enables targeted delivery via ligands or polymers, improving therapeutic efficacy	Complex modifications may increase production costs and potential immunogenicity
Nano-precipitation	Facilitates high drug loading; suitable for small particle sizes	Biodegradable into non-toxic byproducts, such as lactic and glycolic acid, that are eliminated naturally	Rate of biodegradation depends on polymer composition (ratio of lactide to glycolide)
Solvent diffusion	High encapsulation efficiency	High encapsulation efficiency improves drug stability and reduces dose frequency	Drug leakage during preparation may reduce efficacy

CRC treatment is particularly challenged by multidrug resistance (MDR) mechanisms, including efflux transporter upregulation (e.g., ABCB1), defective apoptosis, and tumor heterogeneity. PLGA nanoparticles help bypass these mechanisms by enhancing intracellular drug accumulation, enabling ligand-mediated active targeting (e.g., EpCAM, CD44), and triggered drug release within the tumor microenvironment. In resistant CRC models, PLGA nanocarriers have been shown to restore drug sensitivity by shielding drugs from efflux and modulating immune pathways [16]. Through pulsatile release, PLGA NPs can mimic the effects of multiple injections, boosting immune responses, preventing tumor growth, and extending survival without requiring repeated injections. This improves patient compliance and lowers the risk of treatment-associated metastasis [17].

In addition, PLGA allows surface modifications (e.g., aptamers, antibodies, hyaluronic acid), enabling tumor-specific accumulation and reduced systemic side effects key for chemo resistant metastatic CRC cases. As highlighted in later sections, this has shown promise in animal models with improved survival and immune reactivation. We believe that continued optimization of polymer architecture (e.g., PLA:PGA ratio and surface charge) and integration with stimuli-responsive systems will further improve PLGA-based interventions for chemo resistant CRC [18].

## Mechanism of targeted therapy using PLGA nanoparticles

In order to treat cancer, drugs are often administered orally or systemically, both of which carry the risk of substantial side effects due to their serious accumulation off-target and damage to healthy tissues. Unintended accumulation in non-target areas reduces the allowable dosage of drugs. A potential approach to resolving this issue is the creation of delivery systems that ensure precise targeting of medications.

### Passive targeting

The accumulation of nanocarriers in tumours is largely attributed to the enhanced permeability and retention (EPR) effect. This phenomenon arises from the irregular and permeable tumor vasculature combined with insufficient lymphatic drainage [19]. This phenomenon allows for passive accumulation of NPs in the tumor microenvironment.

PLGA nanoparticles, known for their biocompatibility and tunable degradation, are ideal for such applications. By modifying the surface properties and controlling particle size, PLGA NPs can be optimized for prolonged circulation and enhanced tumor localization [20, 21]. These NPs have been employed to deliver chemotherapeutic agents like doxorubicin (DOX), paclitaxel (PTX), cisplatin, and curcumin (CUR). However, limitations such as tumor heterogeneity, elevated interstitial pressure, and variable vascular permeability affect EPR effectiveness. Understanding tumor pathophysiology remains critical to optimizing passive targeting [22].

### Active targeting

Active targeting involves the functionalization of nanoparticles with ligands that specifically bind to overexpressed receptors on cancer cells, facilitating cellular internalization. These ligands include aptamers, antibodies, peptides, folic acid, vitamin B7, and galactose, among others [23]. Ligand-conjugated PLGA NPs exhibit enhanced cellular uptake, receptor-mediated endocytosis, and selective tumor delivery, thereby improving therapeutic efficacy and reducing off-target effects.

In CRC-specific applications, common receptors targeted include EpCAM (via aptamers), CD44 (via hyaluronic acid), CEA (via scFv antibody fragments), P-selectin (via fucoidan), folate receptors (via folic acid), and ASGPR (via galactose). For instance, docetaxel (DTX)-loaded PLGA NPs modified with poly(dopamine) and TPGS achieved enhanced tumor inhibition. Nevertheless, variability in receptor expression and immune clearance (e.g., via the mononuclear phagocyte system) limit active targeting efficiency. Therefore, ligand selection and tumor profiling are critical to achieving optimal outcomes [24–26].

### Stimuli-responsive targeting

Stimuli-responsive targeting leverages specific internal or external cues to trigger drug release from PLGA nanoparticles, allowing spatial and temporal precision in colorectal cancer therapy. Internal stimuli include tumor-associated features such as acidic pH, elevated glutathione (GSH) levels, and overexpressed enzymes like matrix metalloproteinases (MMPs), while external stimuli encompass magnetic fields, near-infrared (NIR) light, and thermal energy [27]. pH-sensitive PLGA-based systems have been used to enhance camptothecin and curcumin release in acidic tumor environments, whereas redox-sensitive nanoparticles utilize disulfide linkers that degrade in response to high GSH concentrations. Magnetic targeting is recognized as a form of external stimulus-responsive targeting. Applying an external magnetic field results in the aggregation of magnetic NPs at the tumor location [28]. Magnetic targeting has little effect on tumor cell penetration or internalization, but it does lead to nanoparticle accumulation at the tumor cell location. Superparamagnetic iron oxide-loaded PLGA nanoparticles can be guided to tumor sites using external magnets and enhanced by RGD ligand conjugation, enabling both targeted delivery and real-time imaging via Magnetic Particle Imaging (MPI) [29, 30]. Additionally, photothermal PLGA systems loaded with agents like indocyanine green (ICG) can initiate immunogenic cell death and synergize with immunotherapy under NIR light exposure. These intelligent delivery systems not only improve tumor accumulation and reduce systemic toxicity but also hold potential for multimodal cancer treatment strategies [31].

## Recent developments in PLGA-based targeted delivery strategies for colorectal cancer

**Fig. 2:**
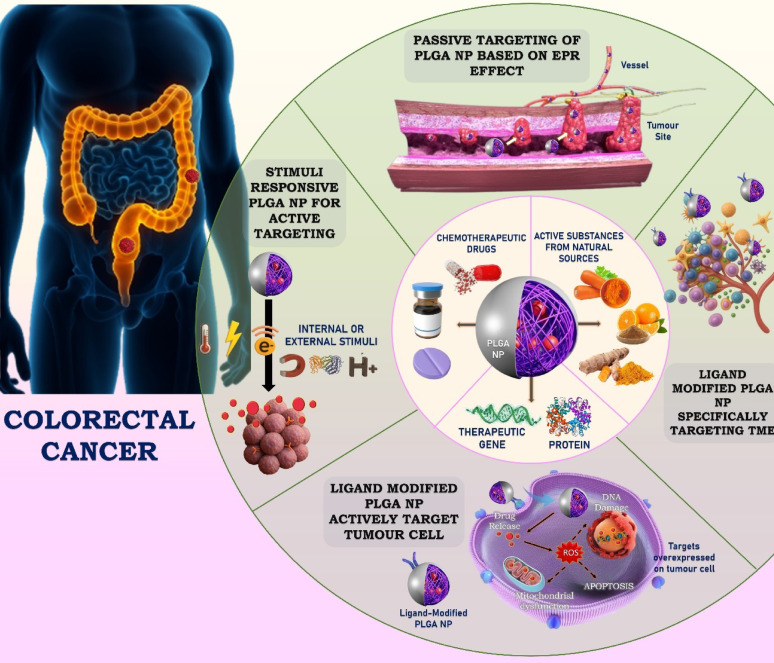
Multimodal strategies using PLGA nanoparticles for precise targeting and enhanced therapy of colorectal cancer

Recent advancements in drug delivery systems have revolutionized CRC treatment, with PLGANPs emerging as a leading platform for targeted therapeutic interventions. This section delves into the state-of-the-art developments in strategies using PLGANPs, focusing on their molecular mechanisms, applications, and clinical implications in CRC treatment (Table 2, Fig. 2).

**Table 2:** Advanced PLGA-based nanoparticle systems delivering diverse therapeutics to colorectal cancer models

Target/mechanism	Payload	Cell line/animal model	Model and key outcome	References
*Strategy: Active Targeting*				
EpCAM/Aptamer-mediated uptake	2c (betulinic acid analogue)	HT-29 and HCT-116/Sprague–Dawley (SD) rats and Swiss albino mice	Aptamer-conjugated PLGA nanoparticles improved betulinic acid analogue delivery to colorectal tumors, enhancing bioavailability, immune activation, tumor specificity, and therapeutic efficacy in vivo compared to non-conjugated formulations	[41]
EpCAM/Aptamer-mediated uptake	5-fluorouracil	HCT-116, CT-26 and HEK-293/BALB/c mice	EpCAM-targeted 5-FU-loaded nanoparticles reduced systemic toxicity, delayed tumor progression, and outperformed free 5-FU, offering a promising approach for colorectal cancer therapy	[42]
CD44/Hyaluronic acid (HA)- mediated uptake	Dactolisib	HCT-116	Hyaluronic acid-conjugated PLGA nanoparticles encapsulating the mTOR inhibitor Dactolisib demonstrated superior cytotoxicity in colorectal cancer cells, effectively disrupting mTOR signaling and overcoming free drug limitations through targeted CD44 receptor delivery	[48]
CD44/Hyaluronic acid (HA)- mediated uptake	β- carotene and 5-Fluorouracil	HCT116(p53-deleted) and uL3ΔHCT 116 ( silenced for uL3)	A novel combination of β-carotene and 5-FU, delivered via HA-based PLGA nanoparticles, counteracted multidrug resistance in colorectal cancer by suppressing ABC transporter expression, enhancing intracellular 5-FU levels, and inducing apoptosis	[49]
CEA	5-Fluorouracil	HCT15, HCT116, HT29, RKO, SW480, SW48, Caco-2 and LS174T	CEA-targeted PLGA/PEG nanoparticles with scFv fragments enhanced tumor specificity, increasing 5-FU uptake and cytotoxicity in colorectal cancer cells while minimizing off-target effects	[56]
P-selectin/Fucoidan‐mediated bindingP-selectin/Fucoidan‐mediated binding	Sorafenib	HCT116 and VERO	Fucoidan-functionalized PLGA nanoparticles improved sorafenib delivery to P-selectin-expressing colorectal tumors, enhancing ROS production, mitochondrial damage, and apoptosis with minimal non-cancer toxicity	[61]
CXCR4	Regorafenib	HCT116	CXCR4-functionalized PLGA nanoparticles co-loaded with regorafenib and radiolabels effectively inhibited tumor growth, targeting metastatic colorectal cancer cells while reducing systemic toxicity	[64]
*Strategy: passive targeting*				
Mucoadhesive and charge-enhanced cellular uptake	5-fluorouracil	HT-29 cells	Chitosan-coated PLGA nanoparticles prolonged drug release, enhanced 5-FU delivery, and improved cytotoxicity against colorectal cancer cells through tailored surface modifications	[66]
PEGylation- and chitosan-based surface modulation	Doxorubicin (DOX), Punicalin (A), and Punicalagin (B)	HCT116 and Caco-2	Chitosan–polyethylene glycol (CS-PEG)-decorated PLGA nanoparticles loaded with ellagitannin demonstrated ROS-mediated cell cycle arrest and enhanced apoptosis in colorectal cancer cells	[67, 68]
pH-responsive passive targeting	Camptothecin	SW-620	Core–shell lipid-polymer nanoparticles enhanced camptothecin stability, controlled release, and selective uptake, improving cytotoxicity and pharmacokinetics for colorectal cancer therapy	[69]
Passive targeting	Enzalutamide	HCT116/Rats	HCT116/Rats; Polysarcosine and TPGS-coated PLGA nanoparticles enhanced enzalutamide bioavailability, reduced toxicity, and improved systemic safety, offering a promising strategy for CRC treatment	[71]
Passive targeting with sustained antioxidant release	Hesperidin	HCT116	PLGA nanoparticles effectively delivered hesperidin, overcoming bioavailability challenges, significantly reducing cancer cell viability, and ensuring controlled drug release in colorectal cancer models	[72]
*Strategy: biomimetic camouflage*				
Homologous active targeting	Etoposide	CT26 cell/Balb/c mice	Cancer cell membrane-coated etoposide-loaded PLGA nanoparticles enabled homologous targeting, enhanced immunogenicity, and inhibited colorectal tumor growth with improved tumor-specific accumulation	[79]
Biomimetic immune evasion and prolonged circulation	Shikonin	HCT116/Male Balb/c mice	RBC-coated PLGA nanoparticles with shikonin improved circulation time, tumor targeting, and antitumor efficacy through immune evasion and enhanced cellular uptake	[80]
Dual targeting: Mannose-mediated TAM (tumor-associated macrophage) targeting + RBC membrane-based immune evasion	Artesunate and Chloroquine	HCT116,CT26 and RAW264.7/Male Kunming mice	Dual-targeting PLGA nanoparticles loaded with artesunate and chloroquine inhibited tumor growth and reprogrammed tumor-associated macrophages in a colorectal cancer mouse model	[81]
Tumor cell membrane-based homologous targeting + immunostimulation	Gambogic acid	CT26 cells and Raw264.7 cells/BALB/c mice	CT26 cells and Raw264.7 cells/BALB/c mice; CCM-PLGA/GA nanoparticles, incorporating gambogic acid and cancer cell membranes, enhanced anti-tumor immune responses, dendritic cell maturation, and synergy with anti-PD-1 therapy in colorectal cancer models	[82]
Membrane-camouflaged targeted delivery	Doxorubicin (Dox) and miRNA-190-Cy7	HCT116, SW480, LS174T, HeLa, HepG2, U87, NCM460, and HUVEC/HCT116xenograft tumor-bearing nude mouse	Membrane-coated PLGA-b-PEG nanoparticles co-encapsulating miRNA-190 and doxorubicin suppressed tumor angiogenesis and growth, enhanced drug sensitivity, and targeted colorectal cancer cells effectively	[83]
*Strategy: stimuli-responsive & smart release*				
Bacteria-mediated tumor targeting + photothermal-triggered immune activation	Cytokine LIGHT	Female BALB/c mice	The photosensitive E.coli-based PLGA/ICG nanoparticle system induced tertiary lymphoid structure (TLS) formation, activating adaptive immunity and inhibiting colorectal cancer growth	[85]
Passive targeting with photo-triggered ROS release and pyroptosis	Curcumin	CT26 and HT29/Female BALB/c mice	PLGA-encapsulated curcumin nanoparticles triggered ROS-mediated pyroptosis via caspase-3/GSDME activation, enhancing programmed cell death and enabling in vivo imaging in colorectal cancer	[86]
Galactose receptor-mediated active targeting	Capecitabine (CAP) and Thymoquinone (TQ)	HT-29 cells/Male Wistar rats	Galactosylated pH-responsive PLGA nanoparticles co-delivered Thymoquinone and Capecitabine, improving therapeutic efficacy, reducing side effects, and targeting colorectal cancer with enhanced precision	[89]
*Strategy: chemo-immunotherapy*				
Macrophage phenotype modulation + immune checkpoint synergy	Retinoic acid	CT-26 and RAW 264.7/Female C57BL/6 J mice	Retinoic acid-loaded cholesterol-coated PLGA nanoparticles combined with anti-PD-L1 therapy modulated tumor-associated macrophages and enhanced antitumor immune responses in colorectal cancer	[96]
AEAA-mediated active targeting	Teniposide	CT26 and CT116/male BALB/C mice	Teniposide-loaded, AEAA-targeted PEGylated PLGA nanoformulation activated the cGAS-STING pathway, inducing apoptosis and promoting chemotherapeutic and immunotherapeutic effects in colorectal cancer models	[97]
Dendritic cell (DC)-mediated antigen presentation and CD8 + T cell activation	Colorectal cancer tumor cell lysates (TCL) and Astragalus polysaccharides (APS)	MC38 and DC 2.4/C57BL/6 mice	LGA-based nanovaccine delivering Astragalus polysaccharides and tumor cell lysates enhanced dendritic cell activation, CD8 + T cell response, and colorectal cancer growth inhibition with good biocompatibility	[98]
*Strategy: gene-silencing*				
Functional targeting via siRNA-mediated Notch1 gene silencing	si-Notch1	HCT8, HCT8-R, SW620 and SW620-R/Females BALB/c nude mice	PLGA nanoparticles carrying si-Notch1 reversed 5-FU resistance in colorectal cancer cells by suppressing glycolysis and inducing pyroptosis	[99]
*Strategy: enzyme therapy*				
Dual targeting	Chloroquine phosphate (CQP), Glucose oxidase (Gox	HCT116/Male Balb/c mice	GOx and chloroquine-loaded biomimetic PLGA nanoparticles increased intracellular ROS, reducing glucose levels, and exhibited synergistic antitumor effects in colorectal cancer models	[106]

### Active targeting (ligand-receptor mediated)

Active targeting has emerged as a powerful strategy to enhance drug accumulation at tumor sites via receptor-ligand interactions. In this subsection, we highlight recent ligand-directed approaches employing PLGA nanocarriers in CRC therapy, showcasing how PLGA’s tunable surfaces and payload versatility make it uniquely suitable for functionalization with aptamers, polysaccharides, and antibody fragments [30].

#### EpCAM-targeted nanoparticles (via aptamers)

Nucleic acid aptamers (Apts), derived through the Systematic Evolution of Ligands by EXponential enrichment (SELEX) process, are short, single-stranded DNA or RNA sequences capable of folding into complex three-dimensional structures, allowing them to bind with high specificity and affinity to their molecular targets [33]. Compared to conventional antibodies, aptamers offer several benefits, including smaller molecular size, reduced risk of immune reactions, improved tissue penetration, simpler and more cost-effective synthesis, and enhanced stability within the body [34]. These features make aptamers promising candidates for use in both cancer diagnostics and targeted therapies [35–38]. One notable aptamer, targeting the epithelial cell adhesion molecule (EpCAM), exhibits strong binding affinity toward this 40 kDa transmembrane protein, which is highly expressed in various epithelial cancers including CRC but found at lower levels in normal epithelial tissues [39]. Nearly 98% of CRC cases present with elevated EpCAM expression, making it an attractive target for therapeutic intervention [40]. Taking advantage of this, researchers have engineered EpCAM-specific aptamer-conjugated drug delivery systems to improve the precision of anticancer agents such as 5-fluorouracil (5-FU) and betulinic acid analogues.

**Fig. 3:**
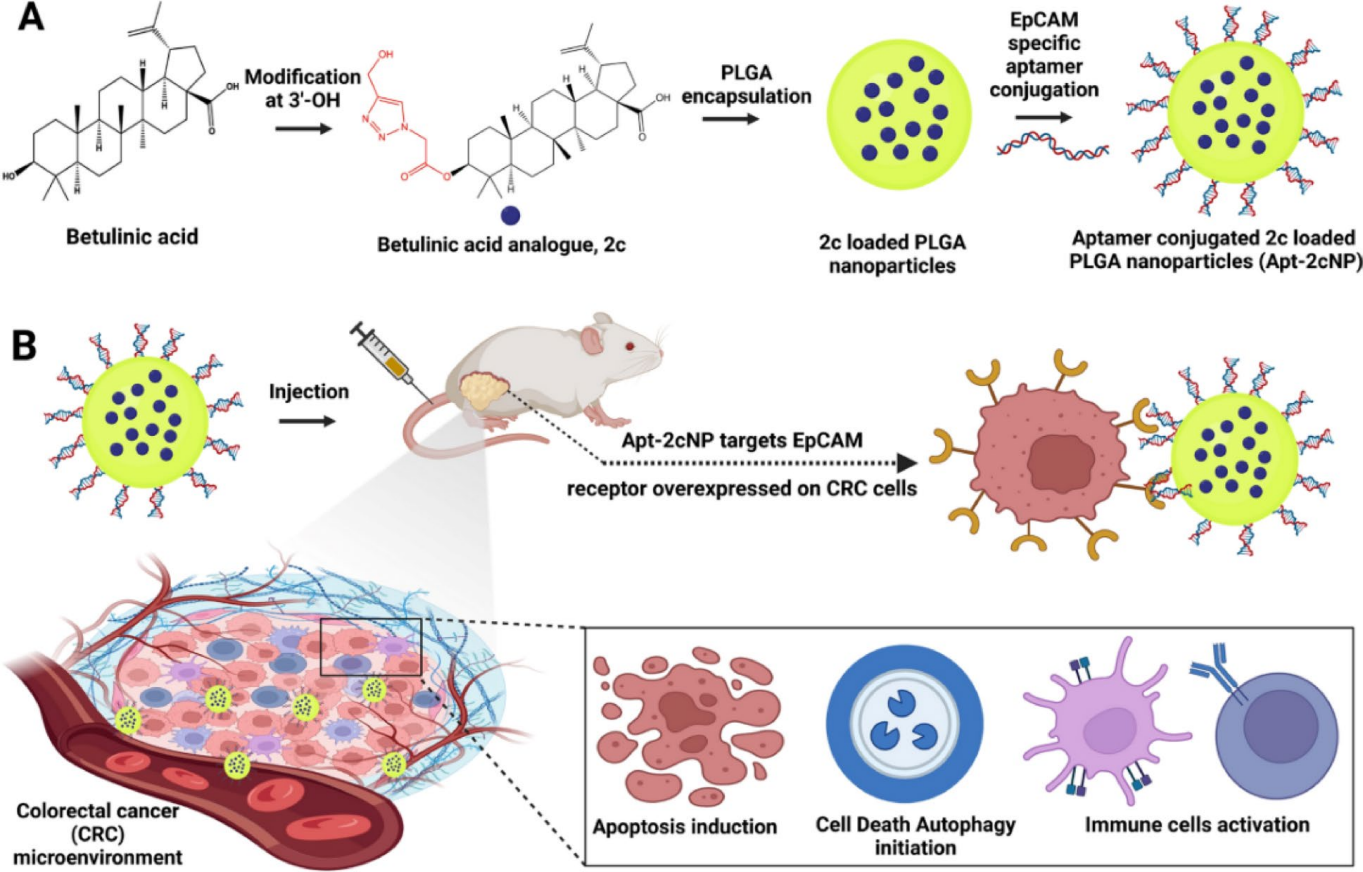
Aptamer-guided PLGA nanocarriers deliver betulinic acid analog to EpCAM-expressing CRC cells, inducing apoptosis, autophagy, and immune activation. Reproduced with permission from [41]

In one approach, Dutta et al*.* developed PLGANPs loaded with a betulinic acid analogue (2c) and modified with EpCAM-targeting aptamers (Apt-2cNPs). In vivo studies demonstrated that Apt-2cNPs not only boosted anticancer activity but also modulated immune responses—elevating mature dendritic cells, M1 macrophages, and cytotoxic CD8 + T cells. Enhanced expression of pro-apoptotic genes and suppression of anti-apoptotic ones further supported their therapeutic superiority over both the free drug and non-targeted formulations (Fig. 3) [41]. Similarly, Yavari et al*.* designed EpCAM-targeted PLGA NPs encapsulating 5-FU (Ap-FU-NPs) to tackle 5-FU’s limitations, including rapid degradation, resistance, and systemic toxicity. These Ap-FU-NPs showed reduced toxicity toward normal HEK-293 cells while demonstrating greater anticancer effects against CT-26 and HCT-116 CRC cell lines compared to non-targeted versions and the free drug. In vivo experiments confirmed their safety profile, as no significant damage to liver, kidneys, or red blood cells was observed. Moreover, tumor growth suppression followed a distinct trend, with Ap-FU-NPs being the most effective, followed by FU-NPs, free 5-FU, and placebo. These results highlight aptamer-functionalized NPs as a promising platform for delivering chemotherapeutic agents more effectively and safely to EpCAM-overexpressing colorectal tumours [42]. Together, these studies illustrate the potential of EpCAM-targeted aptamer-functionalized PLGA NPs to enhance CRC drug delivery, reduce systemic toxicity, and modulate antitumor immune responses. However, further investigation into in vivo targeting efficiency and long-term safety is needed to support clinical translation.

#### CD44-targeted nanoparticles (via HA conjugation)

CD44 is significantly elevated in a range of cancers, including those affecting the breast, lungs, ovaries, brain, and liver. It influences several cancer-related biological functions such as cell differentiation, mobility, blood cell formation, new blood vessel growth, and the spread of tumours [43, 44]. This receptor strongly interacts with glycosaminoglycan compounds like hyaluronic acid (HA) and chondroitin sulphate [45]. HA is a water-attracting, biodegradable, and non-immunogenic polymer naturally present in connective, epithelial, and nervous tissues. When HA binds to CD44, it activates pathways that encourage cancer cell growth, making CD44 a valuable target for anti-cancer strategies [46, 47].

Despite progress, there is still a significant gap in the literature regarding the synthesis and thorough characterization of a drug delivery system (DDS) that includes hyaluronic acid (HA) as a targeting ligand for CD44 receptors, PLGA as the nanocarrier, and a mTOR inhibitor. To overcome this gap, using HA-conjugated PLGA NPs to encapsulate the mTOR inhibitor Dactolisib, Hassan et al*.* developed a targeted delivery method. A measurement of 87.6% for drug encapsulation efficiency showed that Dactolisib was successfully loaded into the NPs. Cytotoxicity studies in vitro showed a substantial decrease in cell viability after DDS treatment. Due to effective delivery and mTOR signaling pathway disruption, the DDS showed more cytotoxicity against CRC cells than free Dactolisib [48].

A significant obstacle in the effective clinical use of 5-FU is the onset of acquired multidrug resistance (MDR), which typically arises during ongoing treatment. Enhancing 5-FU’s therapeutic potential while overcoming drug resistance remains a key challenge in cancer therapy. Carotenuto et al*.* proposed an innovative treatment method targeting MDR in CRC by co-delivering β-carotene and 5-FU using PLGANPs. Their approach was tested on a resistant CRC cell line lacking the p53 gene and with suppressed uL3 expression. Resistance in these cells was primarily caused by the upregulation of ATP-binding cassette (ABC) transporters, which actively remove 5-FU from the cells, reducing its cytotoxic impact. To counter this, the NPs were modified with hyaluronic acid (HA) for targeted delivery via CD44 receptor-mediated uptake, ensuring efficient drug accumulation within cancer cells. β-carotene played a key role by downregulating ABCB1, ABCC9, and ABCC11 transporter gene expression, increasing intracellular 5-FU levels, inducing G2/M phase arrest, and promoting programmed cell death [49]. HA-functionalized PLGA NPs targeting CD44 show promising results in overcoming multidrug resistance in CRC by improving intracellular drug accumulation and modulating transporter gene expression. These findings support their potential in resistant CRC models, though clinical relevance requires validation in large-scale in vivo systems.

#### CEA-targeted nanoparticles (via scFv antibodies)

In approximately 90–95% of primary colorectal tumours and their metastatic counterparts, carcinoembryonic antigen (CEA); a membrane-bound glycoprotein, is found to be highly expressed [50]. This tumor-associated marker plays a critical role in the diagnosis, prognosis, and therapeutic targeting of CRC [51]. Given its tumor-specific expression, CEA has become a key focus for therapeutic intervention, leading to the development of various monoclonal antibodies aimed at this antigen [52, 53]. With progress in antibody engineering, specialized antibody fragments like single-chain variable fragments (scFvs) have been created to improve cancer targeting. One such fragment, MFE-23 scFv, has demonstrated specific binding to CEA-expressing colorectal tumors. Its ability to internalize into tumor cells, remain stable in circulation for over four days at body temperature, and bind effectively to anti-CEA immunotoxins makes it highly suitable for drug delivery and imaging applications [54, 55]. Standard chemotherapy using 5-FU often suffers from poor therapeutic response, systemic toxicity, limited drug absorption, low tumor specificity, and resistance. To address these challenges, Silveira and colleagues designed a nanoparticle system composed of PLGA and PEG, chemically conjugated with the MFE-23 scFv fragment to deliver 5-FU to CEA-expressing CRC cells selectively. These targeted NPs showed a threefold improvement in cellular uptake compared to non-targeted controls. The PLGA core was key in stabilizing the conjugated scFv fragment, enabling prolonged circulation and selective tumor accumulation, essential in tumors with high CEA expression. Enhanced cytotoxic effects were observed after 24 and 48 h of treatment, confirming their tumor selectivity. Additionally, evaluation with macrophages derived from donors revealed no significant changes in their viability or polarization, indicating the biocompatibility of the formulation [56]. Targeting the CEA receptor with scFv-functionalized PLGA-PEG nanoparticles offers tumor selectivity and enhanced uptake in CRC. This approach may be especially useful in CEA-overexpressing tumors but requires assessment of antibody fragment stability and immunogenicity in vivo.

#### P-selectin-targeted nanoparticles (via fucoidan)

P-selectin is a type of adhesion molecule that specifically interacts with carbohydrate structures and is stored in platelet α-granules and endothelial cell Weibel-Palade bodies. When these cells become activated, P-selectin is translocated to their outer membrane, where it facilitates the recruitment of circulating immune cells by enabling their rolling and attachment to activated endothelium [57]. Unlike some adhesion molecules, P-selectin is not present on resting platelets or endothelial cells [58], which minimizes unintended interactions with NPs and preserves normal cellular function. Study by Shamay et al*.* revealed that P-selectin is notably upregulated in a range of cancerous tissues, while its expression remains low in non-cancerous, healthy tissues [59]. Fucoidan, a sulfated polysaccharide derived from brown marine algae, has demonstrated a significantly stronger binding ability to P-selectin than its natural counterpart, PSGL-1 [60]. This property has been strategically exploited to improve the targeted delivery of sorafenib (SOR), a multi-kinase inhibitor that impedes tumor growth, suppresses angiogenesis, and induces cancer cell death. However, clinical use of sorafenib is hindered by low solubility, rapid degradation, and insufficient bioavailability.

To overcome these limitations, Zhou and colleagues engineered a delivery system comprising PLGANPs layered with chitosan and fucoidan (SOR-CS-FU-NPs). These NPs significantly enhanced the cytotoxic impact on HCT116 CRC cells—demonstrating eightfold higher effectiveness compared to free sorafenib. Additionally, uptake of fucoidan-functionalized rhodamine NPs was dramatically improved 19 times higher than standard formulations. The novel system also triggered elevated levels of reactive oxygen species and compromised mitochondrial function, resulting in a 7.5-fold increase in apoptosis. Furthermore, cell migration was markedly suppressed, with minimal wound closure observed. Importantly, the system exhibited no toxicity toward normal VERO cells, highlighting its therapeutic selectivity [61].

#### CXCR4-targeted nanoparticles (via CXCR4L ligand)

The CXCR4 receptor, a type of chemokine receptor, is highly prevalent in over 23 human cancers, such as colorectal, breast, prostate, melanoma, and ovarian cancers, while it is scarcely found in healthy tissues. In CRC, elevated expression of CXCR4 in tumor cells is strongly associated with increased metastasis, higher recurrence rates, and reduced overall survival [62]. This knowledge has led to innovative strategies that utilize CXCR4-binding molecules (CXCR4L) to direct therapeutic agents specifically to cancer cells through nanoparticle-based delivery systems [63].

Regorafenib (RGF), a multi-kinase inhibitor approved by the FDA, is often prescribed to patients with metastatic CRC who no longer respond to first-line treatments. However, its clinical use is limited by severe side effects—reported in 93% of patients during a global phase III trial—and the frequent development of drug resistance shortly after therapy begins. To address these limitations, Cruz-Nova and co-researchers developed a novel treatment system using PLGA NPs encapsulating regorafenib, which were then functionalized with CXCR4L and radiolabelled with the therapeutic isotope lutetium-177 (^1^⁷⁷Lu). Animal studies using the HCT116 CRC model showed that the nano system effectively triggered cancer cell death by disrupting the Akt and Erk signaling pathways. In live models, this formulation significantly suppressed tumor growth. Biodistribution analysis revealed clearance predominantly through liver and kidney pathways. CXCR4-targeted PLGA NPs co-functionalized with lutetium-177 and regorafenib exhibit dual therapeutic and diagnostic utility. These targeted systems may offer effective solutions for metastatic CRC, though concerns remain around radionuclide safety and tissue specificity [64].

### Surface-modified PLGA nanoparticles for sustained and CRC-specific passive targeting

In colorectal cancer therapy, the therapeutic efficacy of PLGA nanoparticles can be significantly hampered by rapid systemic clearance and insufficient tumor retention. To address this, PLGA’s chemically versatile surface can be modified with hydrophilic, mucoadhesive, or immune-evasive materials such as PEG, chitosan, or lipid layers to prolong circulation time, reduce off-target effects, and enhance colon-specific drug delivery [65]. These surface modifications have been successfully used to enhance the therapeutic profile and pharmacokinetics of various drugs for the treatment of CRC.

5-fluorouracil’s (5-FU) short half-life, low oral bioavailability, and severe side effects from non-specificity, limit its efficacy. To overcome the limitations of traditional drug delivery, López-Viota et al*.* developed 5-FU-loaded NPs using (CS), a biodegradable and biocompatible polymer. PLGA-NPs were synthesized using a double emulsion technique and coated with CS at concentrations of 1%, 5%, and 10% (w/v) in an acidic solution. Compared to unmodified NPs, CS-coated NPs exhibited extended drug release, increased cytotoxicity against CRC cells, and enhanced cellular uptake, particularly at higher CS concentrations. CS-coated PLGA NPs significantly enhanced cellular uptake and cytotoxicity in CRC cells, with the most potent effects observed at higher chitosan concentrations [66]. These findings highlight how PLGA’s compatibility with cationic polymers like chitosan enables tunable surface charge and enhanced mucosal adhesion attributes particularly valuable for colorectal delivery. Dual polymer surface modifications have also been used in a similar manner to enhance the anticancer activity and cell-specific effects of chemotherapeutics and bioactives derived from plants. Abd-Rabou et al*.* explored the anticancer potential of chitosan–polyethylene glycol (CS-PEG) modified PLGA NPs (NPs) encapsulating ellagitannin in treating human CRC cell lines (HCT 116 and Caco-2*) *in vitro*.* They developed PLGA-CS-PEG NPs loaded with doxorubicin (DOX), punicalin (PN), and punicalagin (PNG) and evaluated their cytotoxic effects using MTT assays across various cell cycle stages (G0, G1, S, and G2). Post-treatment analysis revealed alterations in protein and gene expression associated with the cell cycle. The NPs showed higher efficacy in Caco-2 cells compared to HCT 116 cells. Nano therapy induced an increase in Bax/Bcl-2 ratio, elevated Cas-3 levels, and decreased expression of Bcl-2, PI3k, and NF-ĸB compared to the control. Additionally, reactive oxygen species (ROS) activity, indicated by nitric oxide (NO) levels, significantly increased following treatment. These findings suggest that PN and PNG-loaded NPs effectively induce ROS-mediated cell cycle arrest, presenting a promising therapeutic strategy for CRC cell lines [67, 68].

In addition to polymer blending, integrating lipid and polymer components into hybrid nanocarriers has demonstrated significant promise. Dave et al*.* explored core–shell hybrid lipid-polymer NPs for delivering camptothecin (CPT) in CRC treatment. These hybrid carriers leveraged the biocompatibility of lipids and the structural stability of polymers, creating a system with enhanced drug release control and cellular uptake. Drug release studies revealed a pH-sensitive profile, demonstrating slower release at physiological pH (7.4) and accelerated release at tumor-like pH (5.5). Cytotoxicity assays on the SW-620 colorectal adenocarcinoma cell line revealed significantly higher cancer cell death and uptake for CPT-loaded NPs compared to the free drug. Moreover, in vivo pharmacokinetic evaluations in rats indicated enhanced plasma drug levels, prolonged retention time, and reduced clearance. This research underscores the potential of hybrid lipid-polymer NPs as a targeted, efficient, and innovative platform for CRC therapy [69]. Recent studies have also reported novel polymeric platforms that disrupt tumor metabolic pathways to improve therapeutic performance in CRC, including the development of metal–phenolic-based polymeric nanomedicines [70]. To improve biocompatibility and systemic safety, advanced polymer coatings have been extensively studied. Ezelutamide, a second-generation androgen receptor (AR) inhibitor, has shown promise in extending survival for patients with castration-resistant prostate cancer (CRPC) and its potential in CRC treatment was explored by Shah et al*.* Using a modified three-step nanoprecipitation and self-assembly method, they developed Enzalutamide (ENZ)-loaded PLGA-NPs coated with polysarcosine and D-α-Tocopheryl polyethylene glycol 1000 succinate (TPGS). In vitro cytotoxicity tests revealed that ENZ-PLGA-PSAR-TPGS NPs were significantly more effective at lower concentrations than the plain ENZ drug, indicating enhanced bioavailability and reduced toxicity. In vivo studies in rats demonstrated no hemolysis, platelet aggregation, or acute toxicity, confirming the biocompatibility of the polymeric NPs. Additionally, no organ damage or changes in body weight, feeding, or drinking behaviors were observed. Pharmacokinetic and tissue distribution analyses showed that ENZ-PLGA-PSAR-TPGS NPs were eliminated faster from the body and exhibited lower liver accumulation than ENZ, suggesting reduced CYP enzyme inhibition. Stability assessments over three months under various conditions confirmed the durability of ENZ-PLGA-PSAR-TPGS NPs. Overall, these findings position ENZ-PLGA-PSAR-TPGS NPs as a promising, safer, and more effective approach for CRC treatment compared to ENZ alone [71]. The success of this multi-layered formulation underscores PLGA’s role as a core carrier, offering stable drug encapsulation and synergistic performance when layered with functional polymers. Alongside traditional chemotherapy, naturally derived antioxidants have gained attention for their potential to enhance therapeutic efficiency when integrated into PLGA NPs. Chemotherapy, while effective, often induces adverse reactions such as nausea, vomiting, and immune suppression. Hesperidin, a natural antioxidant, shows promise in reducing these side effects but suffers from limited bioavailability. To address this, Yaghoubi and colleagues developed PLGA NPs encapsulating hesperidin to evaluate their efficacy against HCT116 CRC cells. These NPs were fabricated using a single emulsion-solvent evaporation method. Drug release reached approximately 93% over 144 h. Notably, the greatest reduction in cancer cell viability was observed at a concentration of 10 µg/ml. These outcomes suggest that hesperidin-loaded PLGA NPs offer a promising strategy for targeted CRC therapy by enhancing antioxidant delivery and minimizing cancer cell survival[72].

Collectively, these surface engineering strategies illustrate how PLGA’s physicochemical flexibility enables a modular approach to nanoparticle design. Through strategic surface modifications, PLGA platforms can overcome rapid clearance, increase tumor residence time, and optimize pharmacokinetics advancing their potential as passive targeting systems for CRC therapy.

### Biomimetic membranes-camouflaged PLGA nanoparticles for CRC therapy

**Fig. 4:**
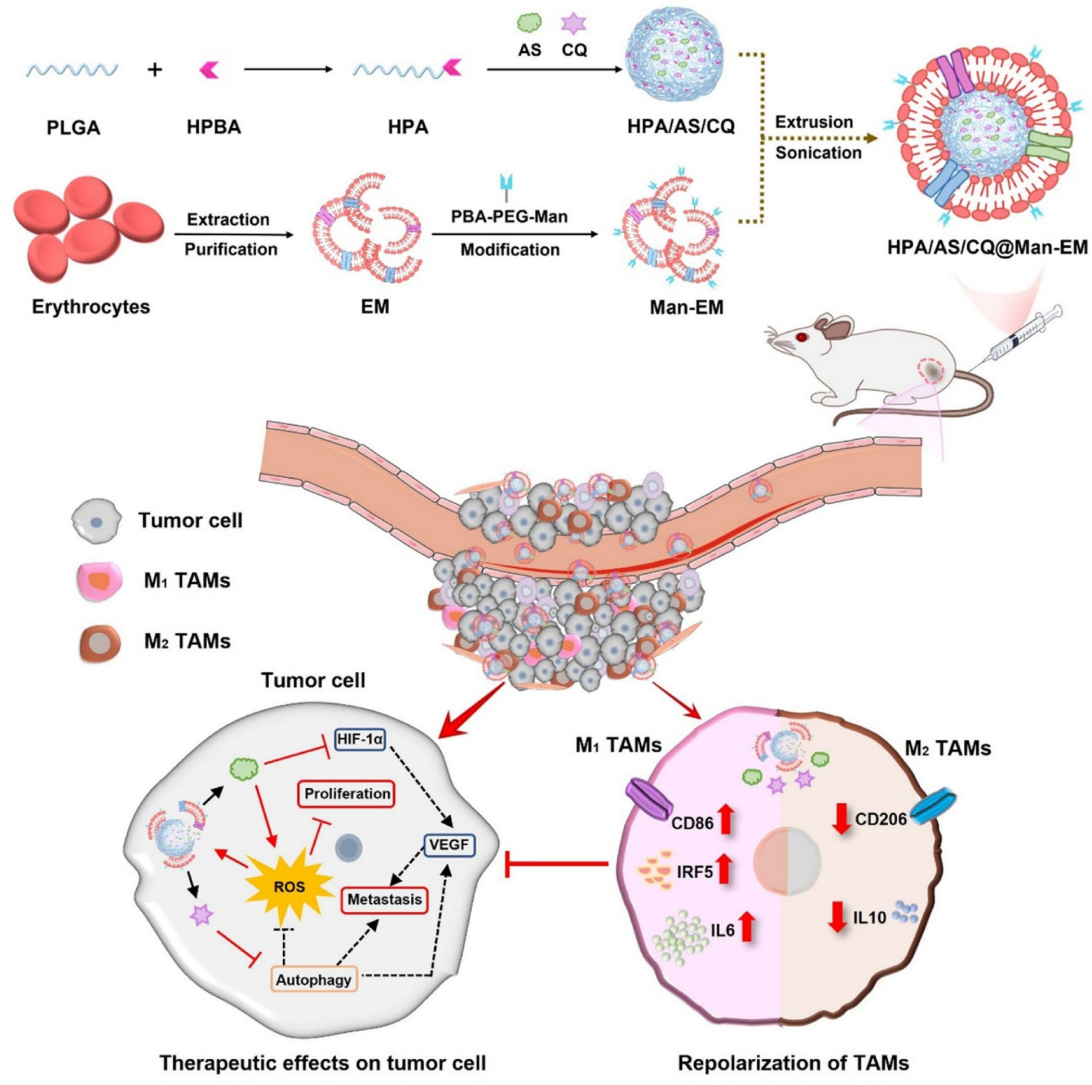
Engineered Man-EM nanoplatform reprograms TAMs and disrupts tumor progression via targeted delivery and immune modulation. Reproduced with permission from [81]

PLGA NPs coated with biological membranes represent a new class of biomimetic nanocarriers with enhanced functionality in colorectal cancer (CRC) therapy. These systems combine the immune-evasive and targeting advantages of natural membranes with the structural integrity, tunable drug release, and biodegradability of PLGA [73, 74]. Various membranes derived from cancer cells, red blood cells (RBCs), or immune cells are used to camouflage PLGA cores, enhancing their circulation time, tumor selectivity, and ability to bypass biological barriers in the tumor microenvironment. Such cell membrane-coated PLGA NPs are particularly advantageous in CRC, where tumor heterogeneity and immunosuppressive signaling reduce conventional drug efficacy [75–79]. RBC membranes have also been used as biomimetic coatings, aiming to combine immune evasion with enhanced drug delivery. It has been reported that "accelerated blood clearance (ABC)" phenomenon the quick removal of PEGylated NPs following the initial injection decreases efficacy by causing the production of anti-PEG antibodies. Zhou et al*.* developed shikonin-loaded PLGA NPs (PLGA/SK) with varied surface charges, utilizing them to design RBC membrane (RBCm)-camouflaged PLGA NPs. Among these biomimetic formulations, negatively charged PLGA NPs exhibited better in vitro stability, as positively charged PLGA NPs tended to affect RBC structure and functionality. However, formulations based on positively charged PLGA NPs demonstrated stronger anti-tumor activity. Compared to RBCm-camouflaged PLGA(+) NPs, RBC-based PLGA(+) delivery systems showed improved tumor targeting in CRC models, prolonged circulation, and higher in vitro cytotoxicity. Overall, RBC delivery vehicles outperformed RBCm-camouflaged systems, offering greater potential for CRC treatment, especially with positively charged NPs. These findings suggest that both surface charge and choice of biomimetic membrane influence immune evasion and therapeutic efficacy in CRC models [80]. Peng et al*.* developed a novel dual-targeting therapeutic approach to address tumor growth and immunosuppressive tumor microenvironments (TME) by utilizing artesunate (AS) and chloroquine (CQ). They synthesized reactive oxygen species (ROS)-sensitive NPs by conjugating hydroxymethyl phenylboronic acid to PLGA, forming the HPA core. This core, containing AS and CQ, was encapsulated with a mannose-functionalized erythrocyte membrane (Man-EM), resulting in biomimetic NPs termed HPA/AS/CQ@Man-EM. These NPs achieved enhanced tumor localization and effectively suppressed tumor progression by disrupting tumor cell proliferation and reprogramming tumor-associated macrophages (TAMs). In an orthotopic CRC mouse model, the biomimetic system demonstrated promising therapeutic effects, offering a potential strategy for advanced cancer treatment (Fig. 4) [81].

Cancer cell membranes have been utilized to create tailored immune-based nano vaccines. Gambogic acid (GA) has shown potential in modulating the tumor immune microenvironment and enhancing anti-tumor therapies. Huang et al*.* designed a nano vaccine to strengthen colorectal cancer (CRC) immune responses by incorporating GA as an immunological adjuvant. GA-loaded PLGA NPs coated with CT26 cancer membranes functioned as personalized nano vaccines, promoting dendritic cell maturation and synergizing with PD-1 blockade in vivo [82]. PLGA served as a stable and immunologically inert backbone for delivering immune-stimulating agents in the nano vaccine format, enabling durable anti-tumor immunity in CRC-bearing mice.

To address challenges in cancer therapy, membrane-camouflaged systems have been utilized for delivering microRNAs (miRNAs) alongside chemotherapeutics. miRNAs, being potential cancer treatment agents, face hurdles such as degradation by RNase, inefficient delivery, limited activity, and uncertain side effects. A versatile and targeted delivery system is crucial to overcome these issues and enhance therapeutic efficacy. In this context, Zhu et al*.* developed membrane-coated PLGA-b-PEG DC-chol NPs (m-PPDCNPs) co-loaded with miRNA-190-Cy7 and doxorubicin (Dox). These NPs exhibited high loading efficiency, minimal toxicity, and superior targeting capabilities. Upon systemic administration in mice, m-PPDCNPs demonstrated selective tumor accumulation. The sustained release of miRNA-190 effectively inhibited tumor angiogenesis, growth, and migration by regulating angiogenic effectors. Additionally, in CRC models, m-PPDCNPs suppressed TGF-β signaling, enhancing the sensitivity of cancer cells to Dox. This innovative approach holds promise for improving therapeutic outcomes by combining miRNA modulation with chemotherapeutic delivery [83]. Overall, cell membrane-camouflaged PLGA nanoparticles offer a versatile and highly CRC-relevant platform for precise drug delivery. The core advantages of PLGA biocompatibility, tunable release, and ligand modification are amplified through biomimetic strategies, enabling deeper tumor penetration, immune evasion, and combination therapy delivery. Such platforms have strong translational potential, especially in overcoming the complex immunosuppressive landscape of colorectal cancer.

### Stimuli-responsive targeting strategies using PLGA nanoparticles in CRC therapy

PLGA NPs have shown tremendous potential in stimuli-responsive cancer therapies, where therapeutic release is activated by internal (e.g., pH, redox, ROS, enzymes) or external stimuli (e.g., light, magnetic fields, heat). In CRC, where site-specific and temporal drug release is critical to overcoming resistance and immune evasion, these smart nanocarriers offer precision in both delivery and therapeutic effect. Unlike other carriers, PLGA allows for fine control over degradation kinetics and stimulus response through its chemical structure and surface modifications [84]. Among these, light-responsive nanocarriers have gained particular attention for their potential in modulating the tumor microenvironment and activating immune responses.

**Fig. 5:**
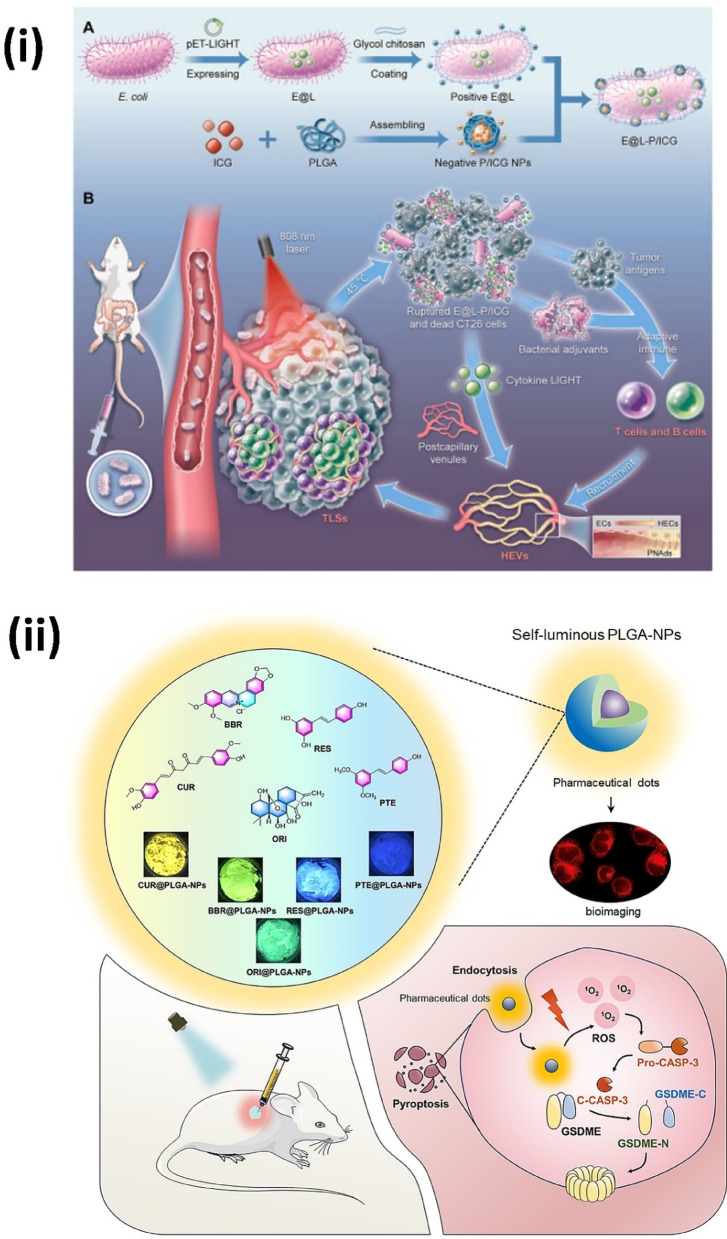
(i) LIGHT-engineered E. coli-coated PLGA nanoparticles activate TLS formation and adaptive immunity for tumor regression upon NIR-triggered photothermal release. Reproduced with permission from [85]. (ii) Self-luminous PLGA nanoparticles encapsulating natural compounds enable real-time tumor imaging and ROS-mediated pyroptosis through GSDME activation. Reproduced with permission from [86]

**Fig. 6:**
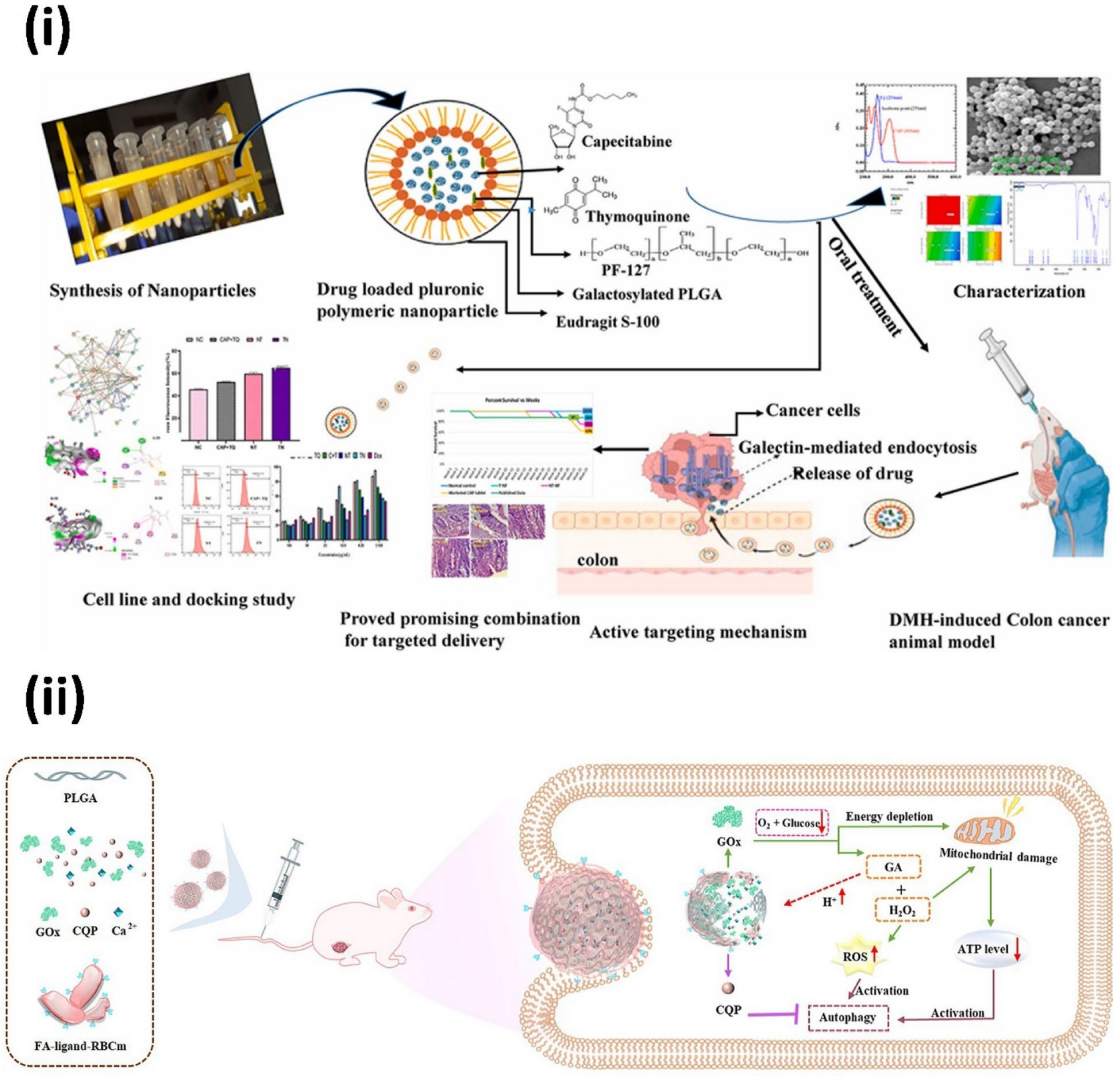
(i) Galactose-decorated pluronic polymeric nanoparticles co-loaded with capecitabine and thymoquinone enable colon-targeted drug delivery via galectin-mediated endocytosis in a DMH-induced cancer model. Reproduced with permission from [89]. (ii) FA-modified RBC membrane-coated PLGA nanoplatform co-delivering GOx, CQP, and Ca^2^⁺ induces starvation-enhanced autophagy and mitochondrial dysfunction for amplified anti-tumor efficacy. Reproduced with permission from [106]

Tertiary lymphoid structures (TLSs) are known to enhance the prognosis of CRC patients by fostering an immune-active tumor microenvironment (TME). Inducing TLS formation therapeutically offers potential for managing immunologically cold CRC, despite the associated challenges. Hu et al*.* developed a photosensitive bacterial platform termed E@L-P/ICG, which integrates E. coli modified with PLGA/ICG (Indocyanine Green) NPs (P/ICG NPs) and loaded with the cytokine LIGHT. In mice with orthotopic colon tumors, E@L-P/ICG accumulates at the tumor site, and upon laser irradiation, the photosensitive P/ICG NPs generate a mild photothermal effect. This effect causes tumor cell death and induces the self-rupture of E@L-P/ICG, releasing antigens and adjuvants that synergistically activate adaptive immune responses. Additionally, the release of LIGHT from the ruptured system stimulates the formation of high endothelial vessels (HEVs), promoting lymphocyte recruitment into the TME. This facilitates TLS formation in CRC, enabling efficient infiltration of T and B cells to enhance adaptive immunity. The combined processes inhibit tumor progression and significantly extend survival in mice. The study highlights the potential of the E@L-P/ICG system to leverage photo-sensitive mechanisms for inducing TLS formation, presenting a promising therapeutic avenue for CRC treatment in clinical settings (Fig. 5-i) [85]. In another approach, photosensitive natural compounds have been used to trigger cancer cell death via reactive oxygen species and programmed pyroptosis. It is frequently noted that natural photosensitizers have a tendency to group together in hydrophilic environments, which makes them vulnerable to aggregation-caused quenching (ACQ) in luminescence and a decrease in ^1^O_2_ generation. Furthermore, in order to be used in CRC treatment, conventional organic fluorophores must be covalently attached to medicinal molecules or carriers, which frequently leads to complicated structures, higher molecular weights, and decreased drug efficacy. Zou et al*.* overcame these obstacles by using PLGA to encapsulate natural substances like curcumin without making any chemical changes, which produced CUR@PLGA-NPs or pharm-dots. When exposed to photoirradiation, CUR@PLGA-NPs were shown to be an efficient ^1^O_2_ producer and to cause pyroptosis in CT26 cells. The activation of the caspase-3/GSDME pathway, as determined by western blotting analysis, demonstrated that ROS produced by CUR@PLGA-NPs may successfully trigger pyroptosis, as demonstrated by CLSM imaging. Additionally, it was noted that CUR@PLGA-NPs’ comparatively strong energy absorption and fluorescence emission properties are set to enhance their exciting potential uses in in vivo imaging (Fig. 5-ii) [86]. In a similar colon-specific strategy, pH-responsive coatings and galactose-mediated targeting have been employed for co-delivery of multiple anticancer agents. Galactosylation increases drug biodistribution in tissues by targeting activated colonic macrophages through galactose receptor-mediated endocytosis [87, 88]. This method has been used to enhance the administration of combination anticancer medications like Thymoquinone (TQ) and Capecitabine (CAP), which is otherwise hindered by a number of factors, including their hydrophobic characteristics, their high first-pass metabolism, their vulnerability to degradation from heat and light, and the notably short half-life of CAP, despite their significant efficacy. Raikar et al*.* developed galactosylated PLGA NPs loaded with CAP and TQ, coated with Eudragit-S-100 (Eud-CAP-TQ-Gal-PLGANP), specifically designed for pH-sensitive drug release in the colon after oral administration. These NPs demonstrated a cell survival rate of 57% at a low concentration of 3.215 μg/mL, with an IC50 value of 4.87. MTT assays and cellular uptake studies revealed significantly enhanced internalization of the targeted NPs into HT-29 cells compared to free CAP and TQ or non-targeted formulations. In vivo experiments conducted on DMH-induced CRC in male Wistar rats confirmed the safety of oral administration, with minimal side effects. Furthermore, TQ exhibited synergistic properties at lower doses, enhancing the anticancer activity of CAP while reducing its adverse effects, highlighting the therapeutic potential of this targeted delivery system (Fig. 6-i) [89].

Stimuli-responsive PLGA nanosystems represent a versatile and intelligent approach to CRC therapy, offering spatiotemporal control over drug release, immunomodulation, and combinatorial strategies such as chemo-photo-therapy or chemo-immunotherapy. Their biocompatibility, structural tunability, and regulatory approval potential position them as key candidates for clinical translation in precision oncology.

### PLGA-based nanoplatforms for combined chemo-immunotherapy in CRC

Conventional chemotherapy causes myelosuppression by having a cytotoxic and cytostatic effect on healthy proliferating cells, particularly hematopoietic cells. This implies that immunotherapy and chemotherapy have an antagonistic relationship. Chemotherapeutics are really certain immunosuppressive medications used to treat autoimmune illnesses or to stop transplant rejection. However, there is growing evidence that the activation of the host immune system plays a significant role in the effectiveness of several cytotoxic medications; in certain situations, these medications may have an immunological stimulatory impact, opening the door for their conjunction with immunotherapy [90–92]. This combination is justified by the fact that immunotherapy can eradicate metastatic and disseminated cancer, but it is less successful at removing a solid tumor mass [93]. This strategy seeks to improve the effectiveness of cancer treatment by concurrently using immunotherapy to strengthen the immune system’s capacity to combat cancer and chemotherapy to target cancer cells [94]. Several nanoparticle-based studies have explored this approach by modulating tumor-associated immune cells or triggering innate immune pathways [95].

Tumor-associated macrophages (TAMs) play a pivotal role in cancer progression by creating an immunosuppressive tumor microenvironment (TME). De Araújo Júnior et al*.* examined whether cholesterol-coated PLGA-NPs carrying retinoic acid, in combination with an anti-PD-L1 checkpoint inhibitor, could mitigate interactions between TAMs and CRC cells. Tumor evaluations utilized qRT-PCR and immunohistochemistry in allograft models, while human tumor samples were analyzed via tissue microarrays and immunohistochemistry. Additional in vitro investigations assessed macrophage polarization, epithelial-mesenchymal transition (EMT), and cell migration. The study revealed that the IL-10R/IL-10 axis suppresses NF-κB signaling and overactivates STAT3, driving the M2-TAM phenotype. Furthermore, cytokines like IL-10, PD-L1, and TGF-β mediate crosstalk between STAT3 and NF-κB pathways through M2-TAMs, exacerbating immunosuppression and EMT in CRC. This activation increases markers such as Vimentin, CXCL12, and CD163 in tumors. PLGA’s ability to encapsulate hydrophobic agents like retinoic acid, while providing cholesterol surface functionality, facilitated macrophage repolarization and enhanced checkpoint blockade response in CRC [96]. Research has explored innate immune sensor activation, such as the cGAS-STING pathway, alongside TAM targeting. To achieve CRC-specific delivery of Teniposide (TEN), Liao et al*.* developed a PEGylated PLGAnanocarrier loaded with TEN, using an AEAA-targeting approach and an oil-in-water emulsion technique. The nanoformulation demonstrated enhanced in vitro anti-CRC effects, including improved cellular uptake, apoptosis induction, antimigration activity, cytotoxicity, and IFN-β production. In a CRC mouse model, the formulation promoted cancer cell death and activated the cGAS-STING pathway, yielding combined chemotherapeutic and immunotherapeutic benefits [97]. Beyond immune pathway activation, another strategy has involved priming dendritic cells and cytotoxic T cells through tumor nanovaccines. In clinical studies, tumor vaccines have shown impressive results in treating patients with a variety of malignancies. However, because of their limited immunogenicity, tumor vaccines have also been shown to fall short of expectations when it comes to treating solid tumors. The development of a tumor vaccination that can elicit a widespread anti-tumor immune response is therefore critically needed. Cao et al*.* designed a nanovaccine (NP-TCL@APS) incorporating Astragalus polysaccharides (APS) and tumor cell lysates (TCL) from CRC into PLGA NPs to trigger a robust innate immune response. Characterization of NP-TCL@APS was performed using transmission electron microscopy and a Malvern particle size analyzer. Its immune activation and cytotoxic effects were evaluated in vitro, followed by safety and anti-tumor efficacy assessments in a CRC mouse model. Results showed that dendritic cells (DCs) efficiently internalized NP-TCL@APS, enhancing DC activation in vitro*. *In vivo, the nanovaccine stimulated antigen-specific CD8 + T cell responses, inhibited tumor growth, and exhibited favourable biocompatibility [98].

Taken together, these studies demonstrate how PLGA’s biocompatibility, co-loading potential, and controlled-release characteristics enable effective convergence of chemotherapy and immunotherapy in colorectal cancer.

### PLGA-mediated gene silencing for reversing chemoresistance in CRC

Gene-silencing using small interfering RNAs (siRNA) offers a powerful approach to overcome chemoresistance in CRC by downregulating key oncogenes. However, successful delivery remains a challenge due to siRNAs short half-life and obstacles in reaching in the target tumor site, making the use of PLGA nanocarriers particularly valuable. Li et al*.* synthesized PLGA NPs encapsulating si-Notch1 and investigated its underlying mechanism and role. It was found that both sensitive and resistant CRC tissues and cells showed high levels of Notch1 expression. Notch1 suppression inhibited glycolysis and encouraged pyroptosis in resistant CRC cells, while also suppressing proliferation and facilitating apoptosis. PCAF has a direct interaction with Notch1. Overexpression of PCAF abolished the glycolysis-suppressive effect of Notch1 knockdown. Additionally, PLGA NPs, a nanocarrier, was developed with a better transport efficiency than lipo2000. In a CRC animal model, Si-Notch1 administered by PLGA NPs effectively overcome the 5-FU resistance of the CRC cells and promoted pyroptosis [99]. This highlights PLGA’s superior performance over conventional lipid-based systems in delivering gene-silencing therapeutics with improved biodistribution, stability, and cellular uptake in CRC models.

### Enzyme therapy

In CRC, enzyme therapy is gaining traction for its tumor-specific catalytic activity and potential to modulate the tumor microenvironment [100]. These include remarkable specificity and affinity for target molecules, along with the ability to catalyse reactions at low concentrations. As a result, research exploring enzyme-based treatments for cancer has expanded [101, 102]. Glucose oxidase (GOx) has emerged as a valuable enzyme that exploits the high glucose metabolism of tumor cells. GOx catalyzes the oxidation of β-D-glucose in the presence of oxygen, producing hydrogen peroxide (H2O2) and gluconic acid. This deprives tumor cells of nutrients while elevating intracellular reactive oxygen species (ROS), disrupting tumor growth [103–105]. Combining GOx with autophagy inhibitors has shown promise, as these inhibitors prevent the removal of H_2_O_2_ via autophagy, enhancing therapeutic effects. Peng et al*.* developed a PLGAmultifunctional nanocarrier co-delivering GOx and chloroquine phosphate (CQP), using a water-in-oil-in-water emulsion to achieve efficient encapsulation and pH-sensitive release via calcium phosphate layers. To enhance targeting, the NPs were coated with red blood cell membranes modified with folic acid. In a mouse model of CRC, the nanocarrier significantly reduced tumor nodules, achieving a 75% reduction compared to free drug combinations. The system’s ability to retain the drug at tumor sites was confirmed through in vivo imaging and biodistribution studies. By simultaneously depleting glucose and increasing ROS levels, this nanocarrier demonstrated a synergistic suppression of tumor progression. This study underscores the irreplaceability of PLGA as a core nanocarrier for enzyme-based combination therapies in CRC, enabling simultaneous metabolic disruption, immune evasion, and tumor-specific targeting (Fig. 6-ii) [106].

## Strategic synthesis: navigating the landscape of PLGA nanocarriers in CRC therapy

The exploration of PLGA nanocarriers in colorectal cancer therapy has unveiled a diverse array of strategies aimed at overcoming two major clinical challenges: chemoresistance and poor drug bioavailability. Of particular interest are PLGA NPs equipped with ligands like EpCAM, CD44, and CXCR4—that guide therapies directly to tumor sites, improving both selectivity and uptake by cancer cells. Another innovative tactic involves camouflaging these NPs with natural cell membranes, such as those from red blood cells or tumor cells themselves. This “disguise” helps them evade detection by the immune system, allowing them to circulate longer in the body and increasing the chances that they reach their target.

Modifying the NP surface, for instance through PEGylation or by creating hybrid structures with both lipids and polymers, further enhances how drugs are carried and released within the body. Recent developments have also focused on stimuli-responsive systems that release their cargo only when triggered by specific conditions like changes in acidity, oxidative environment, or exposure to light which are often unique to tumor tissue. Blending traditional chemotherapy with immunotherapy and gene-silencing tools like siRNA is broadening the therapeutic options, pairing direct tumor attack with efforts to reshape the tumour’s microenvironment.

Despite these advances, bringing these technologies from the lab bench to real-world clinics has been slow, partly due to difficulties in producing these systems consistently at scale, unknown long-term effects, and the lack of universally accepted regulatory standards. Looking ahead, the field needs to focus on developing smart, multi-purpose nanocarriers that can deliver drugs, enable imaging, and even tweak immune responses all in physiologically relevant models and under strict quality control. The progress made so far highlights both the exciting potential and the complexity of turning these scientific advances into truly effective nanomedicines for colorectal cancer.

## Conclusion

When it comes to treating CRC, PLGA NPs have a number of strong benefits. The biocompatible and biodegradable polymer used to make it breaks down into lactic and glycolic acids that the body can eliminate [10, 11]. By modifying PLGA NPs with surface ligands and engineering them for controlled, sustained drug release, it is possible to target tumor cells actively as well as passively (EPR) [18].

According to the studies reviewed here, drug-loaded PLGA NPs significantly enhance delivery. For instance, ligand-conjugated PLGA NPs containing 5-fluorouracil demonstrated greater tumor growth suppression and higher CRC cell uptake than non-targeted or free drug formulations [42, 56, 66]. As numerous reports show improved tumor response and decreased systemic side effects, such formulations increase therapeutic efficacy while reducing off-target toxicity. However, PLGA confronts several obstacles, including low drug loading capacity, difficulty regulating drug release rate in vivo, immunogenicity and immunostimulatory effects on the body, and a lack of long-term stability in vivo. Nonetheless, current advances in synthesis processes and the inclusion of surface changes provide promising paths for addressing these limitations and increasing the therapeutic potential of PLGA [13]. With rigorous preclinical and clinical evaluation, PLGA nanoparticle systems hold strong potential for successful translation from bench to bedside.

## Data Availability

No datasets were generated or analysed during the current study.
